# Synthesis and molecular structure of perhalogenated rhenium-oxo corroles

**DOI:** 10.1038/s41598-020-76308-7

**Published:** 2020-11-12

**Authors:** Abraham B. Alemayehu, Rune F. Einrem, Laura J. McCormick-McPherson, Nicholas S. Settineri, Abhik Ghosh

**Affiliations:** 1 Department of Chemistry, UiT – The Arctic University of Norway, 9037 Tromsø, Norway; 2 Advanced Light Source, Lawrence Berkeley National Laboratory, Berkeley, CA 94720-8229 USA; 3 Department of Chemistry, University of California, Berkeley, Berkeley, CA 94720 USA

**Keywords:** Chemistry, Coordination chemistry, Inorganic chemistry, Organic chemistry, Physical chemistry, Chemical synthesis

## Abstract

As part of our efforts to develop rhenium-oxo corroles as photosensitizers for oxygen sensing and photodynamic therapy, we investigated the potential *β*-perhalogenation of five ReO *meso*-tris(*para*-X-phenyl)corroles, Re[T*p*XPC](O) (X = CF_3_, H, F, CH_3_, and OCH_3_), with elemental chlorine and bromine. With Cl_2_, *β*-octachlorinated products Re[Cl_8_T*p*XPC](O) were rapidly obtained for X = CF_3_, H, and CH_3_, but X = OCH_3_ resulted in overchlorination on the *meso*-aryl groups. Full *β*-octabromination proved slower relative to Cu and Ir corroles, but the desired Re[Br_8_T*p*XPC](O) products were finally obtained for X = H and F after a week at room temperature. For X = CH_3_ and OCH_3_, these conditions led to undecabrominated products Re[Br_11_T*p*XPC](O). Compared to the *β*-unsubstituted starting materials, the *β*-octahalogenated products were found to exhibit sharp ^1^H NMR signals at room temperature, indicating that the aryl groups are locked in place by the *β*-halogens, and substantially redshifted Soret and Q bands. Single-crystal X-ray structures of Re[Cl_8_T*p*CF_3_PC](O), Re[Cl_8_T*p*CH_3_PC](O), and Re[Br_8_T*p*FPC](O) revealed mild saddling for one Cl_8_ structure and the Br_8_ structure. These structural variations, however, appear too insignificant to explain the slowness of the *β*-octabromination protocols, which seems best attributed to the deactivating influence of the high-valent Re center.

## Introduction

The remarkable *β*-octachlorination and *β*-octabromination of metallotetraarylporphyrins was first reported by the Traylor, Dolphin and their research groups in the 1980s^1,2^. During the 1990s, iron and manganese complexes of *β*-octahalogenoporphyrins were intensively investigated as rugged, synthetic models of cytochrome P450^3–6^. *β*-Octahalogeno-*meso*-tetraarylporphyrin derivatives also provided textbook examples of saddling, a nonplanar distortion in which the pyrrole rings are alternately tilted up and down relative to the mean porphyrin plane^7–11^. The compounds became the subject of a battery of spectroscopic, electrochemical, and structural studies, which yielded a rich body of insights on substituent effects in porphyrin derivatives^10,12–15^.

With the advent of simple, one-pot syntheses^16–19^ of corroles^20,21^, *β*-octabromination was also found to work for certain corrole derivatives^22,23^. In our laboratory, we prepared some of the first *β*-octabromo-*meso*-triarylcorroles, initially the copper complexes^24,25^ and subsequently also the free bases^26–28^. Remarkably, a number of crystal structures of *β*-octabrominated metallocorroles revealed planar corrole rings, underscoring the rigidity of the corrole ring system relative to porphyrins^23,29–31^. Today we know that saddling in corroles is largely limited to copper^32–37^ corroles (and in part to silver^38^ corroles but not gold^39–42^ corroles), where it is thought to be a manifestation of ligand noninnocence, i.e., a distortion mode that facilitates Cu^II^(d_x2−y2_)-corrole^⋅2−^ antiferromagnetic coupling^43–46^.

**Figure 1:**
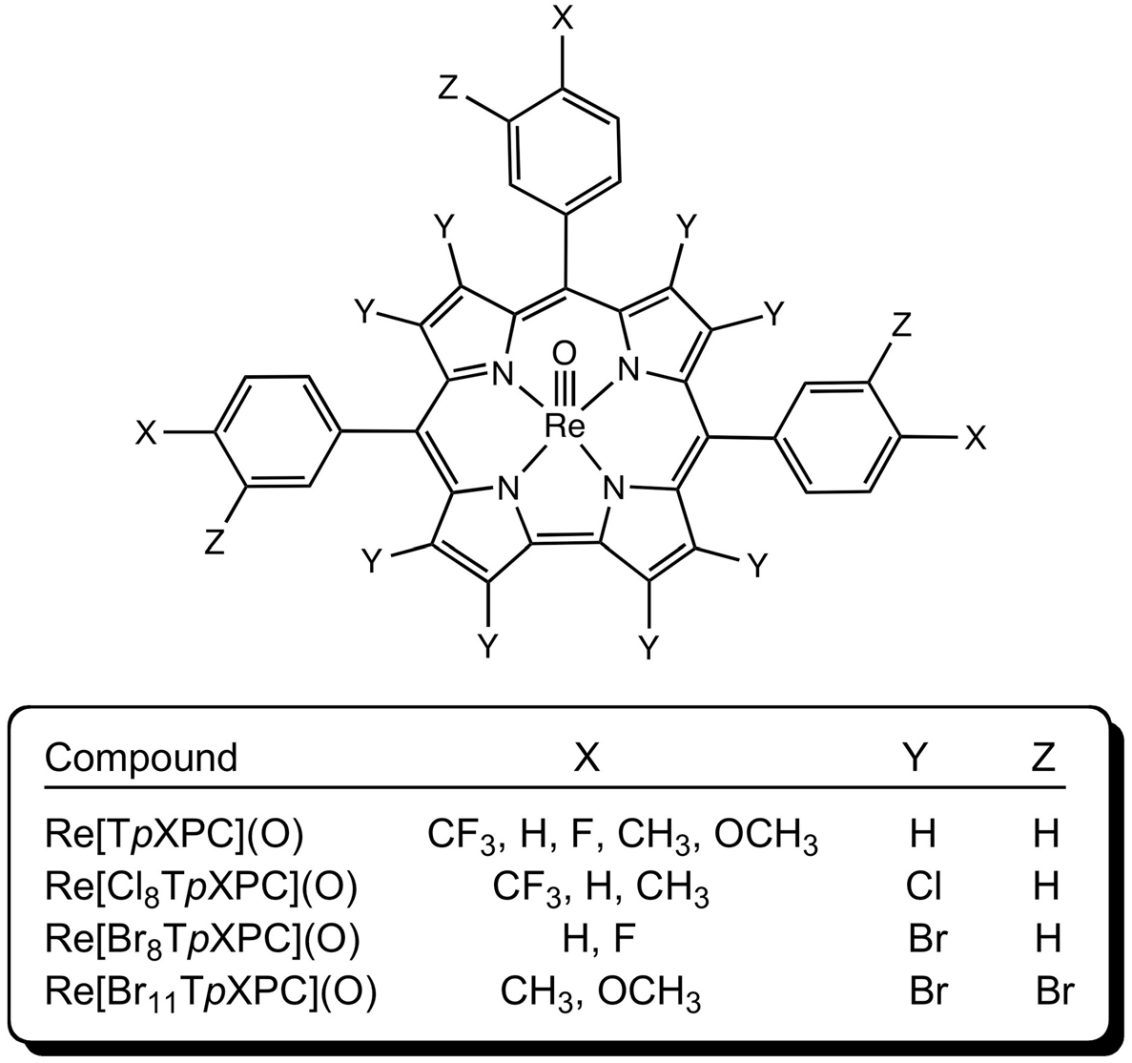
ReO corroles synthesized in this work.

The present study is part of our ongoing efforts to functionalize and derivatize 5d metallocorroles^38–42,47–54^. These complexes provide unusual examples of a large transition metal ion coordinated to a sterically constrained macrocyclic ligand. Despite the steric mismatch inherent in their structures, a good fraction of these complexes exhibit remarkable thermal, chemical, and photochemical stability. Many also exhibit near-IR phosphorescence and also efficiently sensitize singlet oxygen formation, which has led to applications in oxygen sensing, photodynamic therapy, and dye-sensitized solar cells^55–63^. The chemical reactivity of these complexes, by and large, remains poorly explored, with only a handful of reports on the subject. *β*-Octachlorination has been reported for an OsN corrole^64^, while gold corroles have been polyiodinated, with 4–5 iodines attached to the *β*-positions^65,66^. A couple of examples of metal-centered reactivity have also been documented; thus, MoO^67^ and ReO^50^ corroles have been transformed to the MX_2_ (X = Cl, Ph) derivatives, so-called Viking helmet corroles^68^, while OsN corroles have been found to act as unusual π-acceptor metallaligands toward Pt(II)^64^. Herein we document our efforts to halogenate rhenium(V)-oxo triarylcorroles with elemental chlorine and bromine (Fig. 1).

The main contributions of this work may be described as threefold. First and foremost are the products themselves, which should serve as starting materials in a variety of cross-coupling reactions, affording, for example, ReO undecaarylcorroles via the Suzuki–Miyaura reaction. The products thus obtained are likely to further extend the growing range of applications of ReO corroles^60,61^. Second, three of the *β*-octahalogenated products have yielded single-crystal X-ray structures, shedding light on potential distortion modes available to these sterically congested species. Third, although the “messy” reaction conditions did not allow us to devise kinetic studies, qualitative observations indicate major differences in the times required for *β*-octabromination as a function of the coordinated metal, which appear to be ascribable to the electronic effects of the coordinated metal, as described below.

## Results and discussion

### Synthetic method development

Optimizing the conditions for *β*-octachlorination proved relatively straightforward^69^. In the final, optimized protocol, a saturated, greenish-yellow solution of chlorine (Cl_2_) in chloroform was added dropwise, over a period of 20 min, to a benzene solution of *β-*unsubstituted ReO corroles, Re[T*p*XPC](O), maintained at 0 °C in an ice bath. The ice bath was removed after 1 h and the reaction was allowed to continue at room temperature for 24 h. After work-up, HR-ESI mass spectrometry and ^1^H NMR spectroscopy showed that all eight *β-*hydrogens had been fully replaced by chlorine atoms for X = CF_3_, H and CH_3_. For X = OCH_3_, the most electron-donating substituent, however, overchlorination was observed, with Cl_8_, Cl_9_ and Cl_10_ products appearing in a ratio of approximately 15:100:80 (see Figure S11 in the Supplementary Material).

Finding the optimum conditions for *β*-octabromination, in contrast, involved a fair amount of trial and error. Initial experiments with up to 100 equiv liquid bromine (Br_2_) in chloroform led after 4 h to a mixture of Br_4_, Br_5_, Br_6_, and Br_7_ products, with only traces of Br_8_. Increasing the reaction time to 16 h also led to the same complex mixtures. Increasing the reaction time to 48 h, however, led to the selective formation of Br_6_ and Br_7_ derivatives as the major products, with the Br_8_ appearing as a minor product. We surmised that increasing the concentration of elemental bromine and lengthening the reaction time even further might result in full *β*-octabromination. Accordingly, we increased the amount of elemental bromine threefold and extended the reaction time to 7 days. As before, we began with 100 equiv of Br_2_ added dropwise over a 20-min period. On the 2nd day, we added another 100 equiv of Br_2_ dropwise over 20 min. We did the same on the 3rd day and let the reaction run for an additional 4 days, i.e., a total of 7 days. After work up, HR-MS and ^1^H NMR showed that octabrominated complexes Re[Br_8_T*p*XPC](O) had cleanly formed for X = H and F. For X = CH_3_ and OCH_3_, on the other hand, over-bromination had occurred, resulting in the undecabrominated complexes Re[Br_11_T*p*XPC](O).

The long times needed for *β*-octabromination of ReO corroles, and presumably also for OsN corroles, may be contrasted with the rapid reactions observed for Cu^24^ and Ir^23^ corroles. The difference is most simply ascribed to the higher oxidation state of the central metal in the case of the ReO and OsN complexes, which presumably deactivates the corrole toward electrophilic substitution. Such a rationale is in line with the redox potentials of the metallocorroles; the oxidation potentials of ReO^50^ and OsN^52^ corroles are substantially higher than those of analogous Cu^24,38^ and Ir^23^ corroles.

### ^1^H NMR and UV–Vis spectroscopy

**Figure 2:**
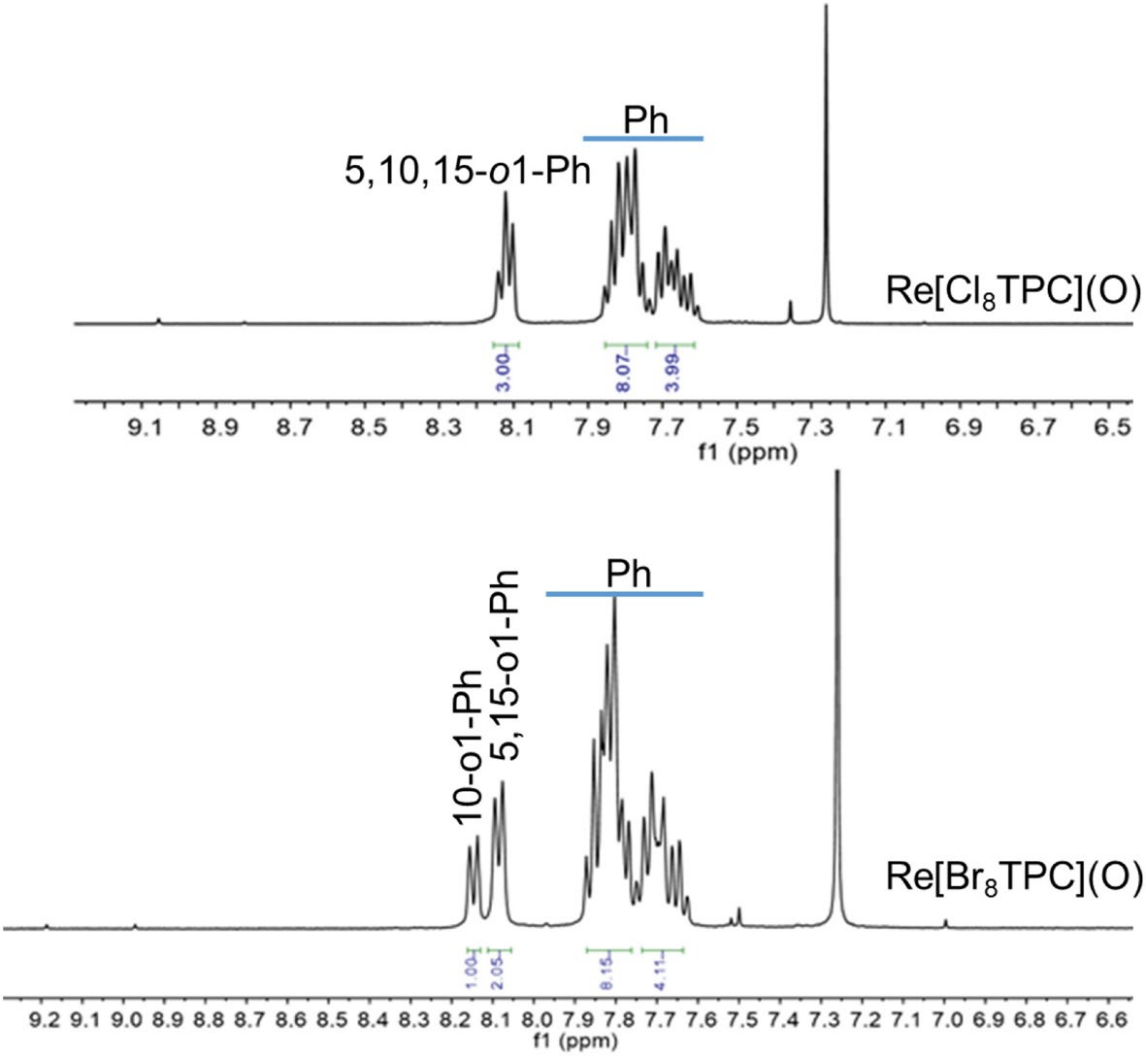
^1^H NMR spectra of Re[Cl_8_TPC](O) (top) and Re[Br_8_TPC](O) (bottom) in CDCl_3_ at 298 K.

Both types of spectra clearly reflect the effect of *β*-octahalogenation. The most obvious change in the ^1^H NMR spectra is associated with the disappearance of the *β*-proton signals between 8.5 and 10 ppm (Fig. 2). Another highly characteristic change is that unlike the room-temperature ^1^H NMR spectra of starting complexes^50^, the spectra of the *β*-perhalogenated products are already sharp at room temperature. The broad ^1^H NMR spectra of Re[T*p*XPC](O) at room temperature reflect partially restricted rotation of the *meso*-aryl groups and only around − 20 °C or so do the aryl *ortho* and *meta* signals split into distinguishable *o*,*o*´and *m*,*m*´signals. In *β*-perhalogenated products, the aryls are effectively locked in place even at room temperature.

**Figure 3:**
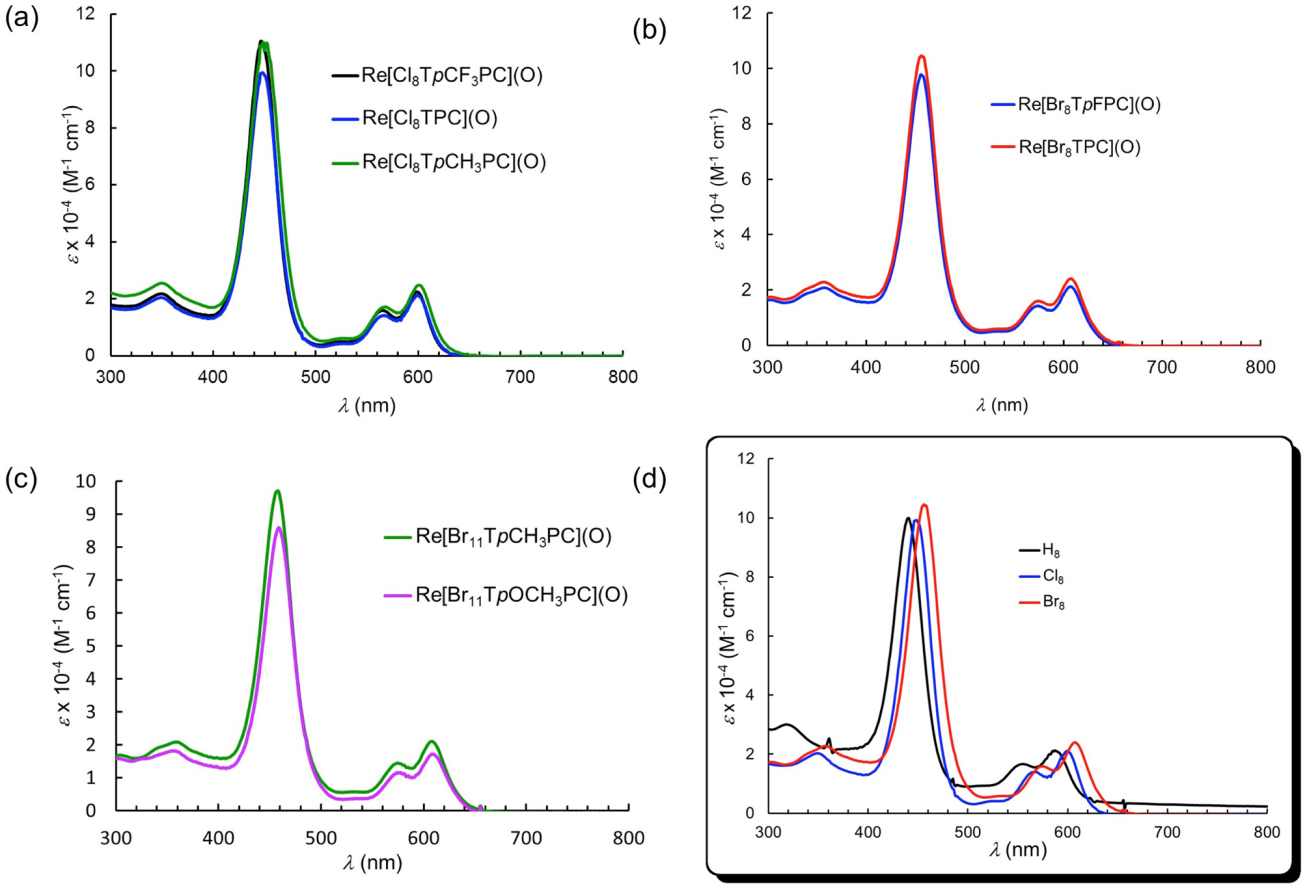
UV–Vis spectra in dichloromethane for (**a**) Re[Cl_8_T*p*XPC](O) (X = CF_3_, H and CH_3_), (**b**) Re[Br_8_T*p*XPC](O) (X = F and H) and (**c**) Re[Br_11_T*p*XPC](O) (X = CH_3_ and OCH_3_). (**d**) Comparative UV–Vis spectra for Re[TPC](O) (black), Re[Cl_8_TPC](O) (blue), and Re[Br_8_TPC](O) (red).

Like a number of other classes of 5d metallocorroles^40,51,52,54^, ReO triarylcorroles exhibit sharp, intense Soret bands and characteristic, double-humped Q bands^50^. The qualitative shapes of these features persist relatively unaltered upon *β*-octahalogenation. *β*-Octahalogenation does, however, engender significant redshifts for each of these features. Thus, for *β*-octachlorination, the Soret and Q bands redshift by around 9 and 13–16 nm, respectively, while for *β*-octabomination, the corresponding shifts are 17–19 and 21–22 nm, respectively (Table 1 and Fig. 3).

**Table 1:** UV–Vis spectral data in dichloromethane, *λ*_max_ and *ε* × 10^−4^ (M^−1^ cm^−1^), for the compounds studied.

Compound	N	Soret	Q_1_	Q_2_	Ref
Re[T*p*CF_3_PC](O)	320 (1.77)	438 (10.74)	552 (1.63)	585 (1.99)	^50^
Re[TPC](O)	320 (1.64)	439 (10.09)	552 (1.99)	585 (2.34)	^50^
Re[T*p*FPC](O)	319 (1.57)	438 (10.16)	553 (1.53)	585 (1.93)	^50^
Re[T*p*CH_3_PC](O)	318 (2.92)	440 (11.18)	555 (1.86)	587 (2.37)	^50^
Re[Cl_8_T*p*CF_3_PC](O)	349 (2.17)	447 (11.04)	565 (1.58)	599 (2.24)	This work
Re[Cl_8_TPC](O)	349 (2.04)	448 (9.93)	567 (1.41)	599 (2.11)	This work
Re[Cl_8_T*p*CH_3_PC](O)	350 (2.54)	449 (11.00)	568 (1.70)	601 (2.47)	This work
Re[Br_8_T*p*FPC](O)	357 (2.08)	456 (9.77)	574 (1.43)	607 (2.12)	This work
Re[Br_8_TPC](O)	357 (2.28)	456 (10.45)	575 (1.59)	607 (2.40)	This work
Re[Br_11_T*p*CH_3_PC](O)	359 (2.07)	458 (9.70)	574 (1.44)	608 (2.10)	This work
Re[Br_11_T*p*OCH_3_PC](O)	356 (1.15)	459 (8.59)	574 (1.15)	608 (1.72)	This work

### X-ray crystallography and molecular structure

The molecular structures of *β*-perhalogenated ReO corroles were expected to be of unusual interest as a window into potential deformation pathways of the corrole macrocycle in response to extreme peripheral crowding^70^. In general, *β*-octahalogenation does not result in significant nonplanar deformations for metallocorroles, reflecting the rigidity of the direct pyrrole–pyrrole linkage^23,28–31^. Coinage metal corroles, especially Cu^32–37^ corroles but also Ag^38^ corroles, constitute the major exceptions to this generalization. These metallocorroles are intrinsically saddled, as a result of a specific metal(d)-corrole(π) orbital interaction, which results in a noninnocent, partial M^II^-corrole^⋅2−^ character of the complexes. Importantly, the degree of saddling in Cu corroles, while substantial even in *β*-unsubstituted triarylcorrole derivatives, can be greatly enhanced by *β*-octasubstitution. The same orbital interaction, however, is energetically unfavorable for Au corroles^40^. Accordingly, even undecasubstituted Au corroles are fairly rigorously planar^29,30,38,40–42^. So, as a matter of fact, are six-coordinate Ir corroles, including Ir *β*-octabrominated derivatives^23,56,62^. Diboron corroles provide another example of a dramatic structural influence of *β*-octasubstitution. Thus, while simple corroles yield strongly domed complexes with *cisoid* FBOBF groups^71^, *β*-octabromo-*meso*-triarylcorroles yield unbridged bis-BF_2_ complexes, with the BF_2_ groups on opposite sides of corrole macrocycle^72^.

**Figure 4:**
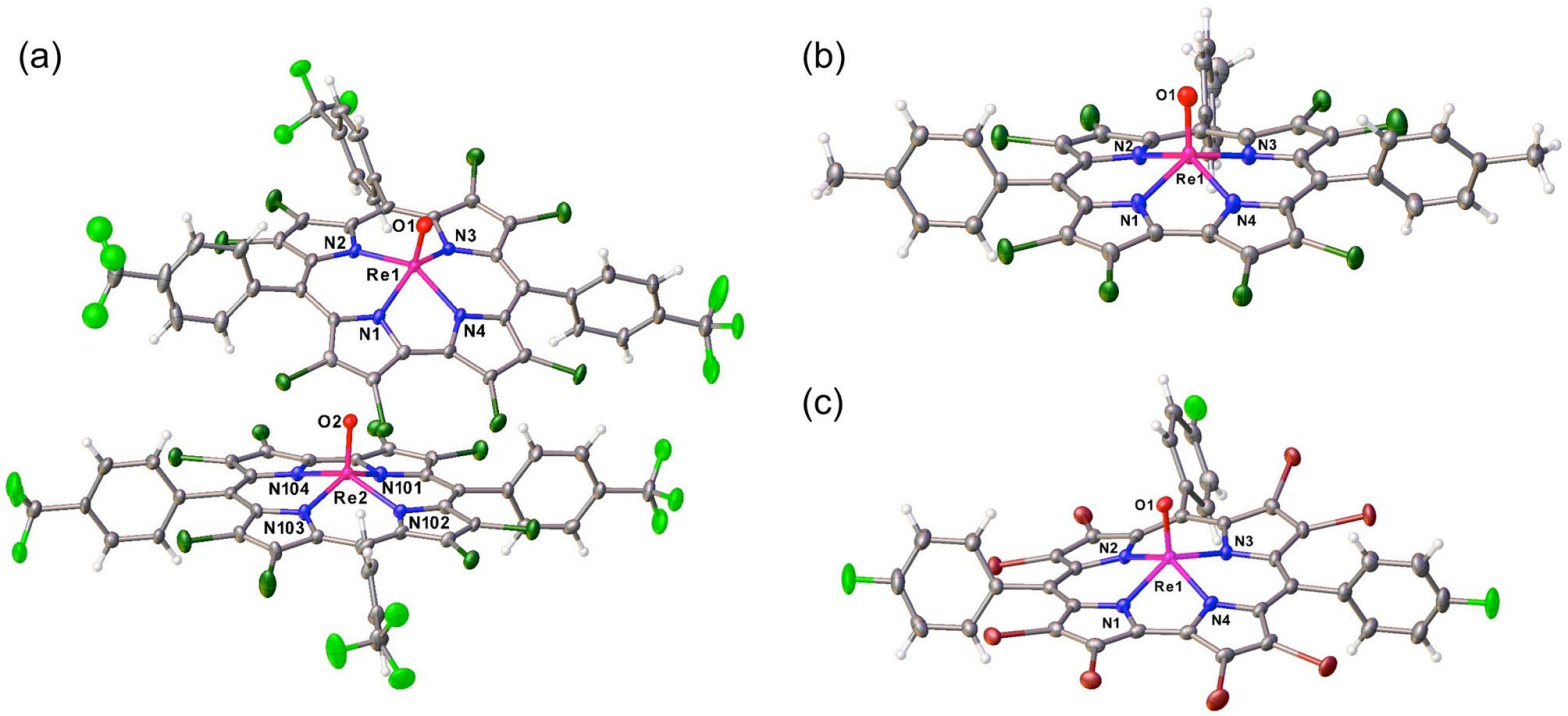
Thermal ellipsoid plots (50%) for (**a**) Re[Cl_8_T*p*CF_3_PC](O), (**b**) Re[Cl_8_T*p*CH_3_PC](O), and (**c**) Re[Br_8_T*p*FPC](O). Selected distances (Å) for (**a**) Re[Cl_8_T*p*CF_3_PC](O): Re1-N1 1.997(5), Re1-N2 2.015(4), Re1-N3 2.025(4), Re1-N4 1.995(4), and Re1-O1 1.670(4); Re2-N101 1.989(4), Re2-N102 2.014(4), Re2-N103 2.019(4), Re2-N104 1.980(4), Re2-O2 1.672. Selected distances (Å) for Re[Cl_8_T*p*CH_3_PC](O): Re1-N1 1.995(3), Re1-N2 2.019(3), Re1-N3 2.017(3), Re1-N4 1.992(3), and Re1-O1 1.677(3). Selected distances (Å) for Re[Br_8_T*p*FPC](O): Re1-N1 1.996(2), Re1-N2 2.019(2), Re1-N3 2.011(2), Re1-N4 1.997(2), and Re1-O1 1.673(2).

**Figure 5:**
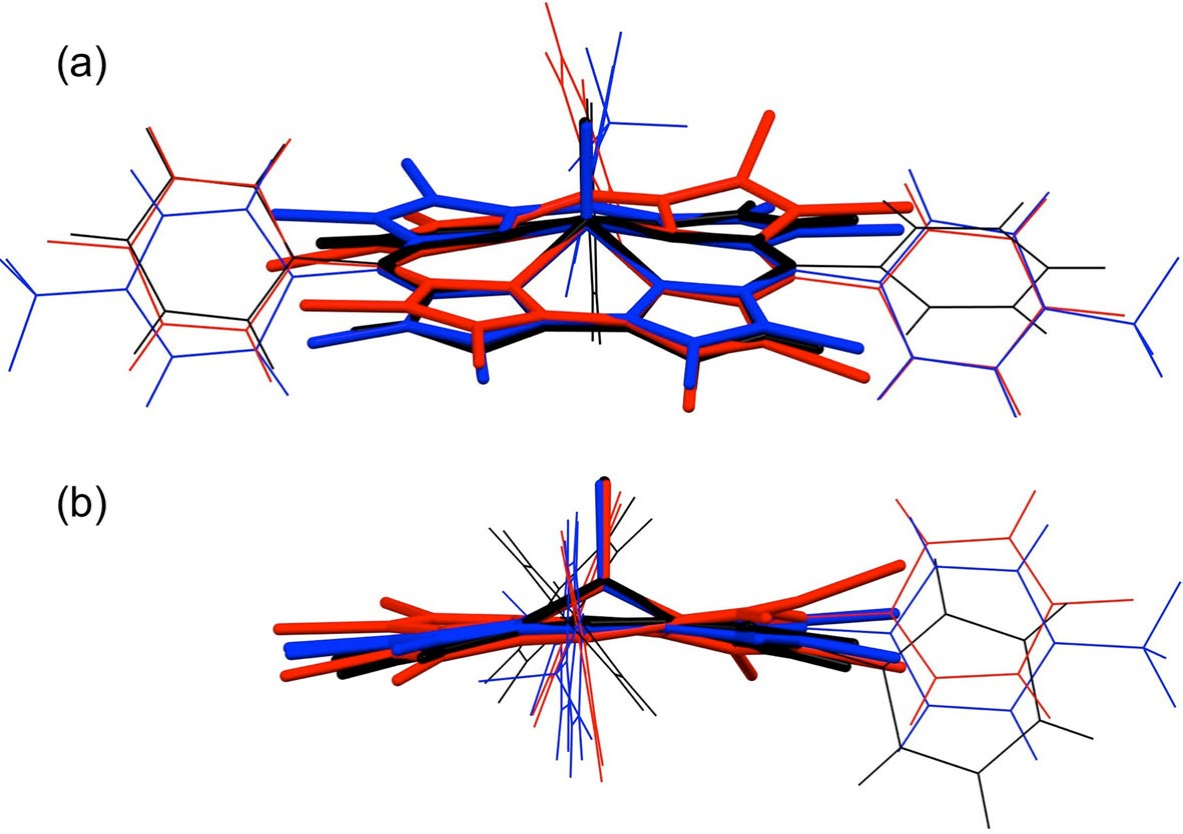
*Mercury* overlay of the nitrogen atoms of Re[TPC](O) (black), molecule 1 of Re[Cl_8_T*p*CF_3_PC](O) (blue) and Re[Br_8_T*p*FPC](O). (**a**) View from above C1–C19 toward C10. (**b**) View along C5–C15.

Three of the products obtained here, including one octabrominated and two octachlorinated products, yielded single-crystal X-ray structures (Table 2). The Re–O and Re–N bond distances, as well as the displacement of the Re atom above the mean N_4_ plane, all turned out to be essentially identical to those observed for *β*-unsubstituted ReO corroles (Fig. 4)^50^. Interestingly, modest variations were observed for the conformations of the corrole macrocycles. Aligning the mean N_4_ planes of *β*-H_8_, *β*-Cl_8_, and *β*-Br_8_ structures showed that the corrole macrocycles in these systems might be described as slightly domed, planar, and slightly, if somewhat irregularly, saddled, respectively (Fig. 5 and Table 3). The *β*-Br_8_ crystal structure reported here thus represents a rare example of a saddled corrole, aside from the coinage metal corroles.

**Table 2:** Crystallographic data for the complexes analyzed.

Sample	Re[Cl_8_T*p*CF_3_PC](O)	Re[Cl_8_T*p*CH_3_PC](O)	Re[Br_8_T*p*FPC](O)
Chemical formula	C_40_ H_12_ Cl_8_ F_9_ N_4_ O Re	C_40_ H_21_ Cl_8_ N_4_ O Re	C_37_ H_12_ Br_8_ F_3_ N_4_ O Re
Formula mass	1203.78	1041.87	1411.38
Crystal system	Triclinic	Triclinic	Triclinic
Crystal size (mm^3^)	0.200 × 0.170 × 0.150	0.050 × 0.030 × 0.010	0.080 × 0.080 × 0.030
Space group	*P*-1	*P*-1	*P-1*
*λ* (Å)	0.7288	0.7288	0.7288
*a* (Å)	16.0327(12)	12.5075(5)	11.7273(4)
*b* (Å)	17.3203(13)	12.9074(6)	14.1820(5)
*c* (Å)	17.8739(13)	13.7640(5)	14.6880(5)
*α* (deg)	116.0080(10)	74.8780(10)	100.8390(10)
*β* (deg)	90.679(2)	85.9610(10)	106.6030(10)
*γ* (deg)	102.117(2)	82.5010(10)	90.7700(10)
*Z*	2	2	2
*V* (Å^3^)	4330.8(6)	2125.22(15)	2293.38(14)
Temperature (K)	100(2)	100(2)	100(2)
Density (g/cm^3^)	1.998	1.765	2.269
Measured reflections	108,021	59,434	151,248
Unique reflections	25,785	8713	11,449
Parameters	1231	567	602
Restraints	59	9	6
*R*_int_	0.0685	0.0511	0.0405
*θ* range (deg.)	1.341–31.393	1.573–27.142	2.027–29.131
*R*_1_, *wR*_2_ all data	0.0780, 0.1432	0.0332, 0.0794	0.0259, 0.0526
*S* (GooF) all data	1.112	1.075	1.039
Max/min res. dens. (e/Å^3^)	2.370/ − 2.057	2.163/ − 1.534	1.714/ − 1.377

**Table 3:** Measures of nonplanarity for Re corroles: the Re–N4 displacement (Å), the C8–C9–C11–C12 saddling dihedral (°), and the C5–Re–C15 angle (°).

Molecule	Re–N_4_ (Å)	χ_C8–C9–C11–C12_ (°)	*ϕ*_C5–Re–C15_ (°)
Re[TPC](O)	0.704	7.2	155.1
Re[Cl_8_T*p*CF_3_PC](O); molecule 1	0.671	15.4	155.1
Re[Cl_8_T*p*CF_3_PC](O); molecule 2	0.659	6.7	154.0
Re[Cl_8_T*p*CH_3_PC](O)	0.671	8.5	157.4
Re[Br_8_T*p*FPC](O)	0.668	11.9	152.5

Might the above structural differences play a role in explaining the slow rates of *β*-octabromination of ReO triarylcorroles relative to Cu and Ir triarylcorroles? Given that the above differences are rather minor (Table 3), we believe that the answer is essentially ‘no’; as stated above, the high oxidation state of the Re center provides the most plausible explanation for the slowness of the octabromination.

## Conclusion

In summary, we have optimized reaction conditions leading to *β*-perhalogenation of ReO triarylcorroles with elemental chlorine and bromine. *β*-Perhalogenation is accompanied by highly characteristic changes in the ^1^H NMR and UV–Vis spectra of the compounds. Three of the *β*-octahalogenated products, including two of the octachlorinated complexes and one octabrominated complex, yielded single-crystal X-ray structures. On the whole, the structures were remarkably similar to those of *β*-unsubstituted ReO corroles. Minor variations were observed in regard to macrocycle conformation. Thus, whereas *β*-unsubstituted ReO corroles are generally slightly domed, one octachlorinated complex and the octabrominated complex were found to exhibit slightly saddled macrocycles. These structural differences, however, appear to be too minor to explain the comparative slowness of *β*-octabromination of ReO triarylcorroles, relative to their Cu and Ir counterparts. The slowness is more plausibly attributed to the high oxidation state of the Re center, which leads to a higher oxidation potential for the corrole macrocycle and, in turn, a lower susceptibility to electrophilic attack. It is hoped that the *β*-perhalogenated complexes reported herein will act as substrates in Suzuki–Miyaura and other palladium-catalyzed transformations, thereby affording additional avenues for the elaboration of ReO corroles.

## Experimental section

### Materials

Rhenium-oxo *meso*-triarylcorroles, Re[T*p*XPC](O), were synthesized as previously reported^50^. Chlorine gas (Cl_2_), liquid bromine (Br_2_), benzene, and chloroform were purchased from Sigma-Aldrich. Silica gel 60 (0.04–0.063 mm particle size, 230–400 mesh, Merck) was used for flash chromatography and silica gel 60 preparative thin-layer chromatography (PTLC) plates (20 cm × 20 cm, 0.5 mm thick; Merck) were used for final purification of all complexes.

### Instrumental methods

UV–visible spectra were recorded on an HP 8453 spectrophotometer. ^1^H NMR spectra (298 K, CDCl_3_) were recorded on a 400 MHz Bruker Avance III HD spectrometer equipped with a 5-mm BB/1H SmartProbe and referenced to residual CHCl_3_ at 7.26 ppm. High-resolution electrospray-ionization (HR-ESI) mass spectra were recorded from methanolic solution on an LTQ Orbitrap XL spectrometer.

### General procedure for the synthesis of Re[Cl_8_T*p*XPC](O)

To a 10-mL benzene solution of Re[T*p*XPC](O) (X = CF_3_: 15 mg, 0.016 mmol; X = H: 25 mg, 0.034 mmol; X = CH_3_: 20 mg, 0.026 mmol) chilled to 0 °C in an ice-bath, was added a greenish-yellow, saturated solution of chlorine (Cl_2_, 10 mL) in chloroform over a period of 20 min. After an hour at 0 °C, the ice-bath was removed and the reaction was allowed to continue under stirring at room temperature for a total of 24 h. The reaction mixture was then quenched by washing twice with 20% aqueous sodium metabisulfite solution (20 mL × 2). The organic phase was thoroughly washed with distilled water, dried with sodium sulfate, and rotary-evaporated to dryness. The resulting crude product was dissolved in a minimum amount of dichloromethane and loaded onto a silica gel column prepared with 3:1 hexane/dichloromethane and eluted with the same solvent system. The resulting greenish-red product was collected, evaporated to dryness, and further purified via preparative thin-layer chromatography with the same solvent system. Yields and analytical details of new compounds are given below. X-ray-quality crystals of Re[Cl_8_T*p*CF_3_PC](O) and Re[Cl_8_T*p*CH_3_PC](O) were obtained by slow diffusion of methanol vapor into concentrated dichloromethane solutions of the complexes.

### Re[Cl_8_T***p***CF_3_PC](O)

Yield 11.5 mg (59.22%). UV–Vis (CH_2_Cl_2_) *λ*_max_ [nm, *ε* × 10^−4^ (M^−1^ cm^−1^)]: 349 (2.17), 447 (11.04), 565 (1.58), 599 (2.24). ^1^H NMR (400 MHz, 25 °C) *δ*: 8.29 (d, 3H, ^3^*J*_HH_ = 7.92 Hz, 5,10,15-*o*1-Ph); 8.09 (d, 2H, ^3^*J*_HH_ = 7.96 Hz, 5,15-*o*2-Ph); 8.05 (d, 1H, ^3^*J*_HH_ = 7.60 Hz, 10-*o*2-Ph); 7.98 (d, 4H, ^3^*J*_HH_ = 6.40 Hz, 5,15-*m*1& *m*2-Ph); 7.92 (d, 1H, ^3^*J*_HH_ = 9.80 Hz, 10-*m*1-Ph); 7.84 (d, 1H, ^3^*J*_HH_ = 8.00 Hz, 10-*m*2-Ph). Elemental analysis calcd for C_40_H_12_ON_4_F_9_Cl_8_Re: C 39.86, H 1.00, N 4.65; found: C 40.27, H 1.21. N 4.24. MS (ESI): M^+^  = 1203.7915 (expt), 1203.7880 (calcd for C_40_H_12_ON_4_F_9_Cl_8_Re).

### Re[Cl_8_TPC](O)

Yield 23 mg (66.71%). UV–Vis (CH_2_Cl_2_) *λ*_max_ [nm, ε × 10^−4^ (M^−1^ cm^−1^)]: 349 (2.04), 448 (9.93), 567 (1.41), 599 (2.11). ^1^H NMR (400 MHz, 25 °C): *δ* 8.12 (d, 3H, ^3^*J*_HH_ = 7.48 Hz, 5,10,15-*o*1-Ph); 7.85–7.73 (m, 8H, Ph); 7.70–7.60 (m, 4H, Ph). Elemental analysis calcd for C_37_H_15_ON_4_Cl_8_Re: C 44.38, H 1.51, N 5.60; found; C 44.07, H 1.37, N 5.18. MS (ESI): M^+^  = 999.8272 (expt), 999.8258 (calcd for C_37_H_15_ON_4_Cl_8_Re).

### Re[Cl_8_T***p***CH_3_PC](O)

Yield 15.3 mg (56.39%). UV–Vis (CH_2_Cl_2_) *λ*_max_ [nm, ε × 10^−4^ (M^−1^ cm^−1^)]: 350 (2.54), 449 (11.00), 568 (1.70), 601 (2.47). ^1^H NMR (400 MHz, 25 °C): *δ* 7.97 (d, 3H, ^3^*J*_HH_ = 8.24 Hz, 5,10,15-*o*1-Ph); 7.64 (d, 2H, ^3^*J*_HH_ = 7.96 Hz, 5,15-*o*2-Ph); 7.60 (d, 2H, ^3^*J*_HH_ = 7.68 Hz, 5,15-*m*1-Ph); 7.55 (d, 1H, ^3^*J*_HH_ = 8.92 Hz, 10-*o*2-Ph); 7.49 (d, 3H, ^3^*J*_HH_ = 7.72 Hz, 10-*m*1& 5,15-*m*2-Ph); 7.43 (d, 1H, ^3^*J*_HH_ = 7.72 Hz, 10-*m*2-Ph); 2.72 (s, 6H, 5,15-*p*-CH_3_); 2.69 (s, 3H, 10-*p*-CH_3_). MS (ESI): Elemental analysis calcd for C_40_H_21_ON_4_Cl_8_Re: C 46.04, H 2.03, N 5.37; found: C 46.11, H 1.67, N 5.60. MS (ESI): M^+^  = 1041.8746 (expt), 1041.8728 (calcd for C_40_H_21_ON_4_Cl_8_Re).

### General procedure for the synthesis of Re[Br_8_T*p*XPC](O) and Re[Br_11_T*p*XPC](O)

To a chloroform solution of Re[T*p*XPC](O) (10 mL; X = F: 8 mg, 0.0102 mmol; X = H: 16 mg, 0.022 mmol; X = CH_3_: 10 mg, 0.013 mmol, and X = OCH_3_: 15 mg, 0.018 mmol) was added a solution of liquid bromine (a total of 300 equiv) solution in chloroform in three 10-mL portions (each containing 100 equiv Br_2_ and added over 20 min) at 24-h intervals over 3 days. The reaction was then allowed to proceed under stirring at room temperature for a total of 7 days. The resulting mixture was quenched by washing with 20% aqueous sodium metabisulfite (25 mL × 3). The organic phase was then washed with distilled water (50 mL), dried over sodium sulfate, and rotary evaporated to dryness. The crude reaction mixture was loaded onto a silica gel column prepared with 4:1 hexane/dichloromethane and eluted with the same solvent system. The greenish-red product was rotary evaporated to dryness and further purified via preparative thin-layer chromatography with the same eluent. Detailed analytical and respective yield of the compounds synthesized are given below. X-ray-quality crystals of Re[Br_8_T*p*FPC](O) were obtained by slow diffusion of methanol vapor into a concentrated benzene solution of the complex.

### Re[Br_8_T***p***FPC](O)

Yield 9 mg (62.09%). UV–Vis (CH_2_Cl_2_) *λ*_max_ [nm, ε × 10^−4^ (M^−1^ cm^−1^)]: 357 (2.08), 456 (9.77), 574 (1.43), 607 (2.12). ^1^H NMR (400 MHz, 25 °C): *δ* 8.11 (m, 1H, 10-*o*1-Ph); 8.05 (m, 2H, 5,15-*o*1-Ph); 7.78 (m, 2H, ^3^*J*_HH_ = 7.68 Hz, 5,15-*o*2-Ph); 7.65 (m, 1H, 10-*o*2-Ph); 7.55–7.36 (m, 6H, 5,10,15-*m*1 & *m*2-Ph). Elemental analysis calcd for C_37_H_12_F_3_ON_4_Br_8_Re: C 31.50, H 0.86, N 3.97; found: C 31.78, H 1.24, N 3.68. MS (ESI): M^+^  = 1411.3885 (expt), 1411.3889(calcd for C_37_H_12_F_3_ON_4_Br_8_Re).

### Re[Br_8_TPC](O)

Yield 20 mg (66.76%). UV–Vis (CH_2_Cl_2_) *λ*_max_ [nm, ε × 10^−4^ (M^−1^ cm^−1^)]: 357 (2.28), 456 (10.45), 575 (1.59), 607 (2.40). ^1^H NMR (400 MHz, 25 °C): *δ* 8.14 (d, 1H, ^3^*J*_HH_ = 7.48 Hz, 10-*o*1-Ph); 8.08 (d, 2H, ^3^*J*_HH_ = 7.40 Hz, 5,15-*o*1-Ph); 7.86–7.62 (m, 12H, Ph). Elemental analysis calcd for C_37_H_15_ON_4_Br_8_Re: C 32.75, H 1.11, N 4.13; found: C 32.49, H 0.95, N 3.88. MS (ESI): M^+^  = 1357.4166 (expt), 1357.4174(calcd for C_37_H_15_ON_4_Br_8_Re).

### Re[Br_11_T***p***CH_3_PC](O)

Yield 12 mg (56.36%). UV–Vis (CH_2_Cl_2_) *λ*_max_ [nm, ε × 10^−4^ (M^−1^ cm^−1^)]: 359 (2.07), 458 (9.70), 574 (1.44), 608 (2.10). ^1^H NMR (400 MHz, 25 °C): *δ* 8.35 (s, 1H, Ph); 8.28 (s, 1H, Ph); 8.02 (s, 1H, Ph); 7.97 (d, 1H, ^3^*J*_HH_ = 7.68 Hz, Ph); 7.92 (d, 2H, ^3^*J*_HH_ = 8.44 Hz, Ph); 7.68 (d, 3H, ^3^*J*_HH_ = 8.08 Hz, Ph); 7.59 (d, 1H, ^3^*J*_HH_ = 7.84 Hz, Ph); 7.53 (s, 1H, Ph); 2.78 (s, 6H, 5,15-*p*CH_3_); 2.72 (s, 3H, 10-*p*CH_3_). MS (ESI): Elemental analysis calcd for C_40_H_18_ON_4_Br_11_Re: C 29.37, H 1.11, N 3.43; found: C 29.59, H 1.41, N 3.33. M^+^  = 1635.1998 (expt), 1635.1936(calcd for C_40_H_18_ON_4_Br_11_Re).

### Re[Br_11_T***p***OCH_3_PC](O)

Yield 19 mg (59.81%). UV–Vis (CH_2_Cl_2_) *λ*_max_ [nm, ε × 10^−4^ (M^−1^ cm^−1^)]: 356 (1.82), 459 (8.59), 574 (1.15), 608 (1.72). ^1^H NMR (400 MHz, 25 °C): *δ* 8.34 (s, 1H, Ph); 8.28 (s, 1H, Ph); 8.01 (s, 1H, Ph); 7.71 (d, 2H, ^3^*J*_HH_ = 8.72 Hz, Ph); 7.57 (d, 2H, ^3^*J*_HH_ = 8.36 Hz, Ph); 7.35 (d, 3H, ^3^*J*_HH_ = 9.32 Hz, Ph); 7.19 (d, 1H, ^3^*J*_HH_ = 8.20 Hz, Ph); 4.23 (s, 6H, 5,15-*p*OCH_3_); 4.18 (s, 3H, 10-*p*OCH_3_). Elemental analysis calcd for C_40_H_18_O_4_N_4_Br_11_Re: C 28.53, H 1.08, N 3.33; found: C 28.17, H 1.35, N 2.98. MS (ESI): M^+^  = 1683.1775 (expt), 1683.1784(calcd for C_40_H_18_O_4_N_4_Br_11_Re).

### X-ray structure determinations

All X-ray diffraction data were collected on beamline 12.2.1 at the Advanced Light Source of Lawrence Berkeley National Laboratory, Berkeley, California. The samples were mounted on MiTeGen kapton loops and placed in a 100(2) K nitrogen cold stream provided by an Oxford Cryostream 700 Plus low temperature apparatus on the goniometer head of a Bruker D8 diffractometer equipped with PHOTONII CPAD detector. Diffraction data were collected using synchrotron radiation monochromated with silicon(111) to a wavelength of 0.7288(1) Å. In each case, an approximate full-sphere of data was collected using 1° *ϕ* and *ω* scans. Absorption corrections were applied using SADABS^73^. The structure was solved by intrinsic phasing (SHELXT)^74^ and refined by full-matrix least squares on *F*^2^ (SHELXL-2014)^75^ using the ShelXle GUI^76^. Appropriate scattering factors were applied using the XDISP^77^ program within the WinGX suite^78^. All non-hydrogen atoms were refined anisotropically. Hydrogen atoms were geometrically calculated and refined as riding atoms.

## Supplementary information

Supplementary Information 1.

## Data Availability

The crystal structures described in this paper have been deposited at the Cambridge Crystallographic Data Centre and been assigned the following deposition numbers CCDC 1532043-1532045.
