# Supplementary material for: Synthesis and molecular structure of perhalogenated rhenium-oxo corroles
Source: Sci Rep. 2020 Nov 12;10:19727. doi: 10.1038/s41598-020-76308-7 (PMC7665048; doi:10.1038/s41598-020-76308-7)
Supplement: Supplementary file 1 — Supplementary Information 1. [file 41598_2020_76308_MOESM1_ESM.pdf]

Supplementary Material

Synthesis and Molecular Structure of Perhalogenated Rhenium-Oxo Corroles

Abraham B. Alemayehu,[1] Rune F. Einrem,[1] Laura J. McCormick-McPherson,[2] Nicholas S. Settineri,[2,3] and Abhik Ghosh*[,1]

[1]Department of Chemistry, UiT – The Arctic University of Norway, N-9037 Tromsø, Norway; Email: abhik.ghosh@uit.no (AG)

[2]Advanced Light Source, Lawrence Berkeley National Laboratory, Berkeley, CA 94720-8229, USA

[3]Department of Chemistry, University of California, Berkeley, Berkeley, California 94720, USA

Table of contents

NMR spectra	S2
ESI mass Spectra	S5

a) NMR Spectra

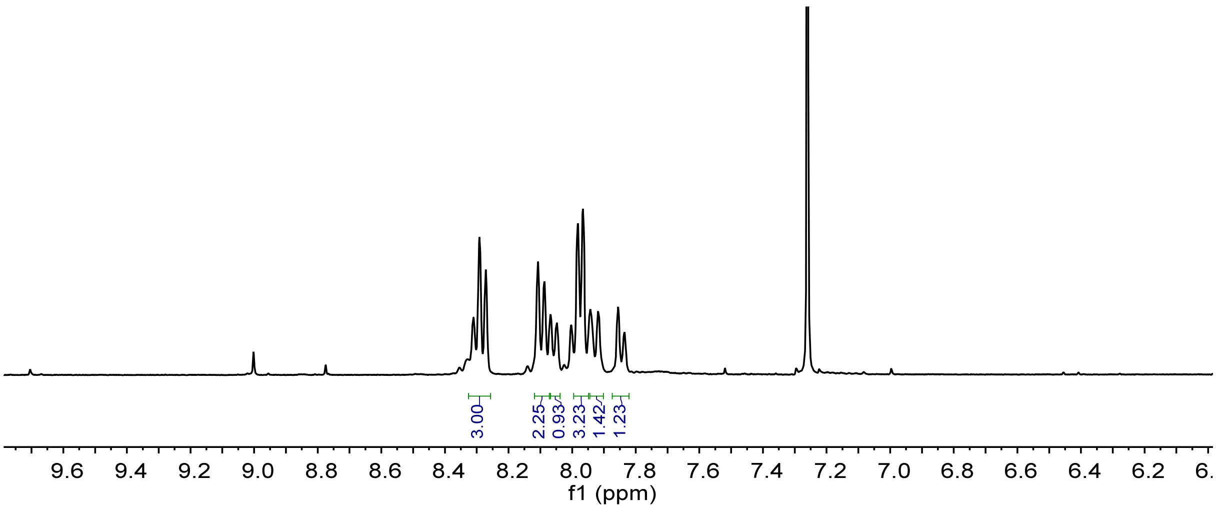

Figure S1. ^{1}H NMR spectrum of Re[Cl_8T*p*CF_3PC](O) in $CDCl_3$ at 298 K.

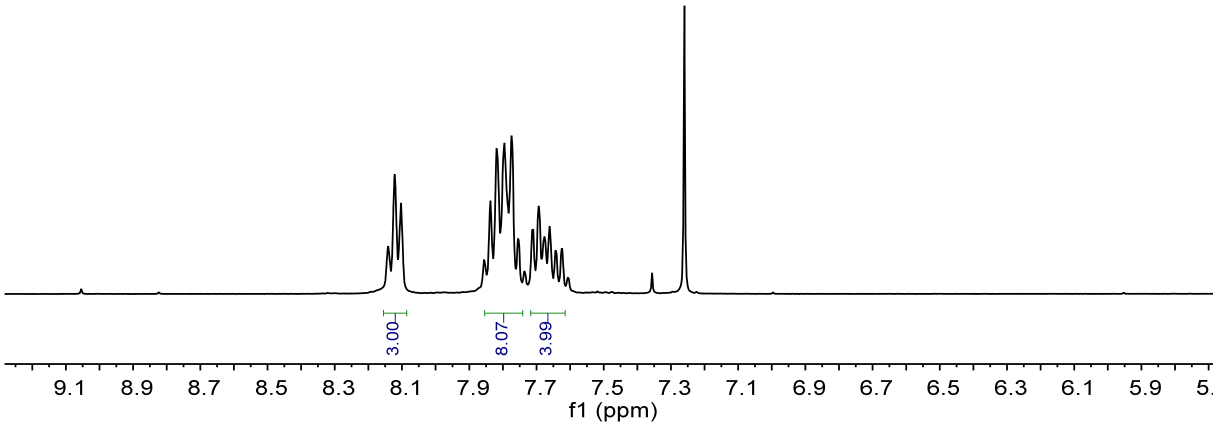

Figure S2. ^{1}H NMR spectrum of Re[Cl_8TPC](O) in $CDCl_3$ at 298 K.

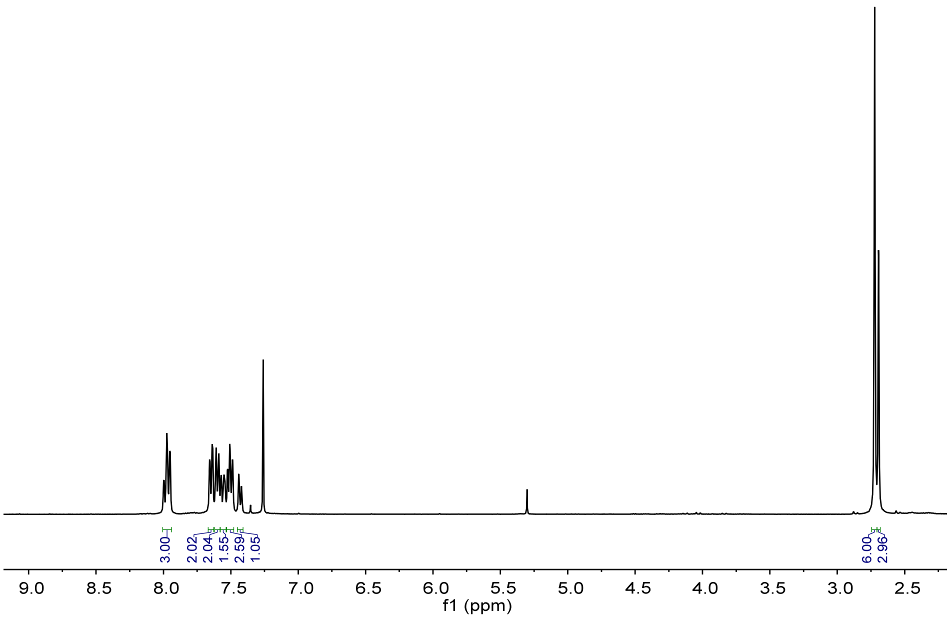

Figure S3. ^{1}H NMR spectrum of Re[Cl_8T*p*CH_3PC](O) in $CDCl_3$ at 298 K.

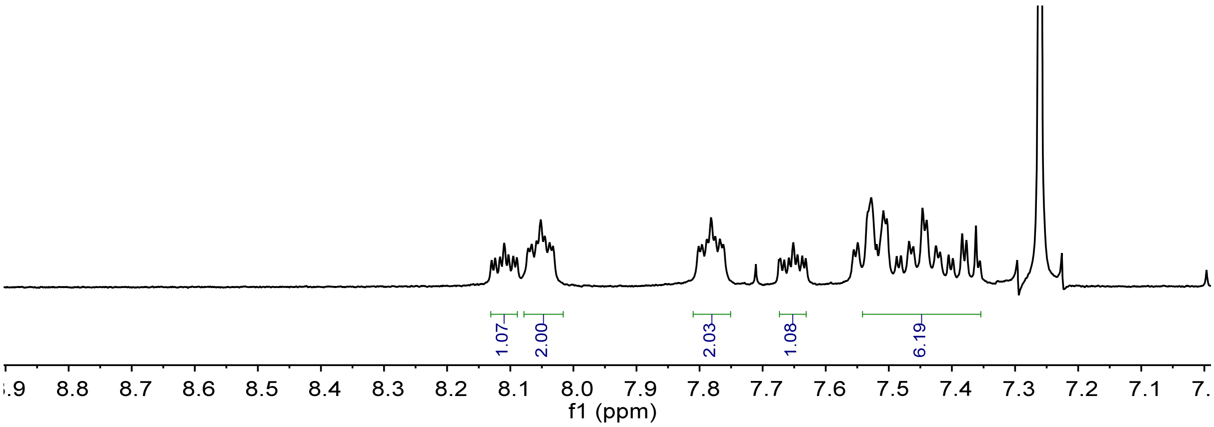

Figure S4. ^{1}H NMR spectrum of Re[Br_8T*p*FPC](O) in $CDCl_3$ at 298 K.

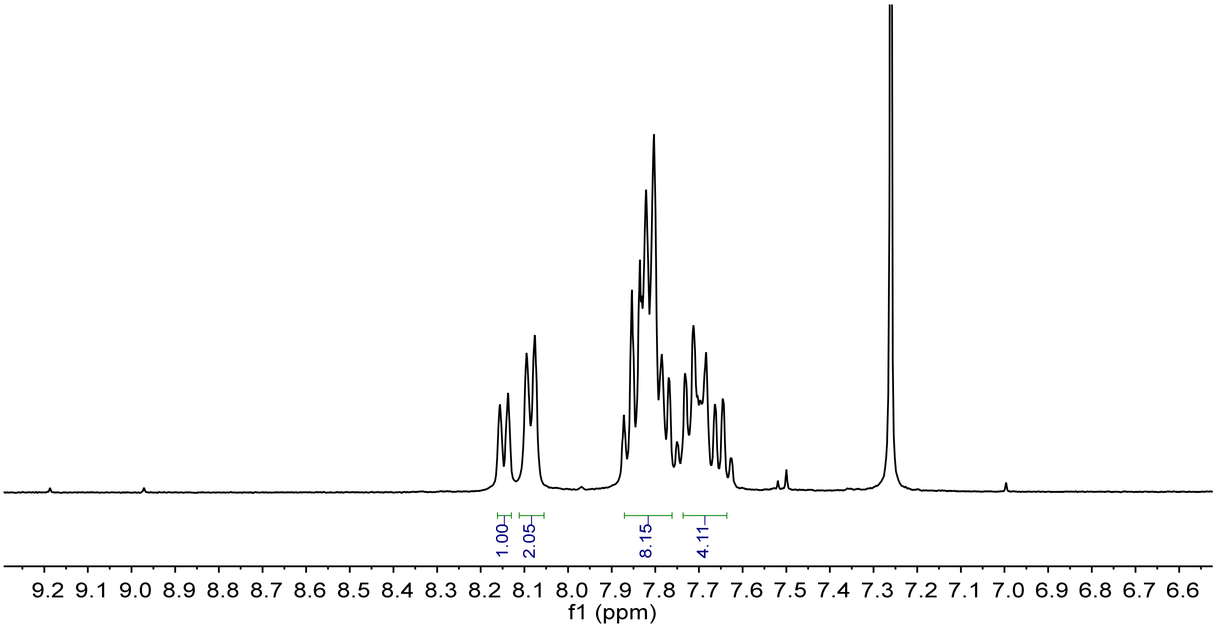

Figure S5. ^{1}H NMR spectrum of Re[Br_8TPC](O) in $CDCl_3$ at 298 K.

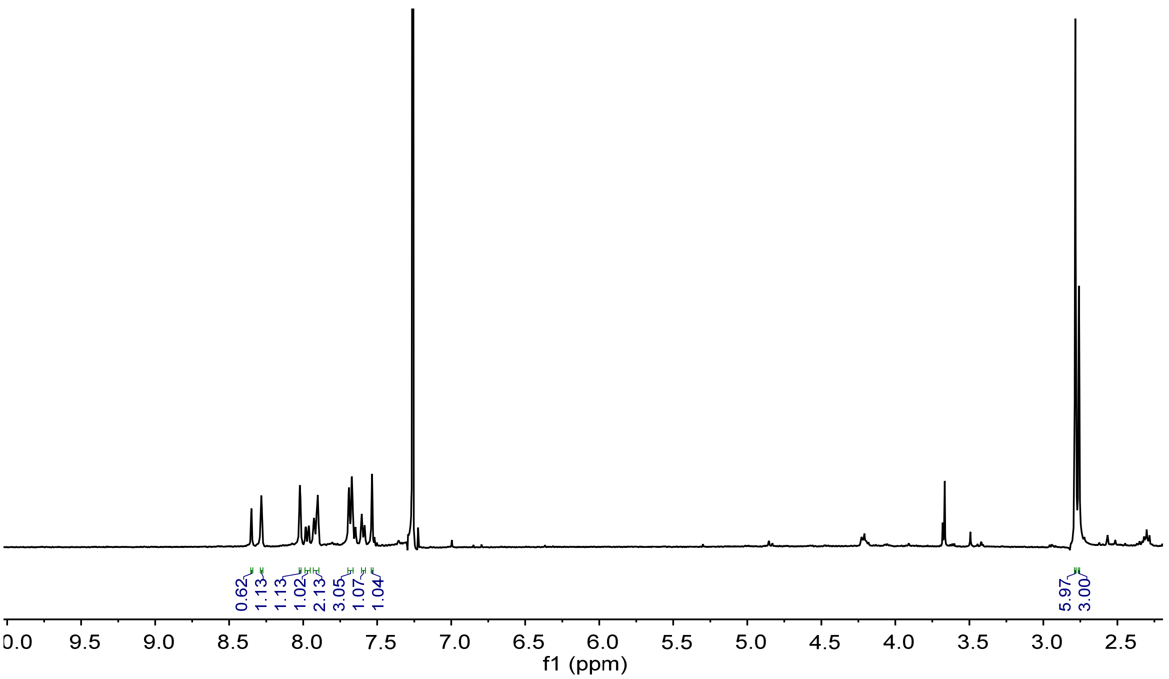

Figure S6. ^{1}H NMR spectrum of Re[Br_{11}T*p*CH_3PC](O) in $CDCl_3$ at 298 K.

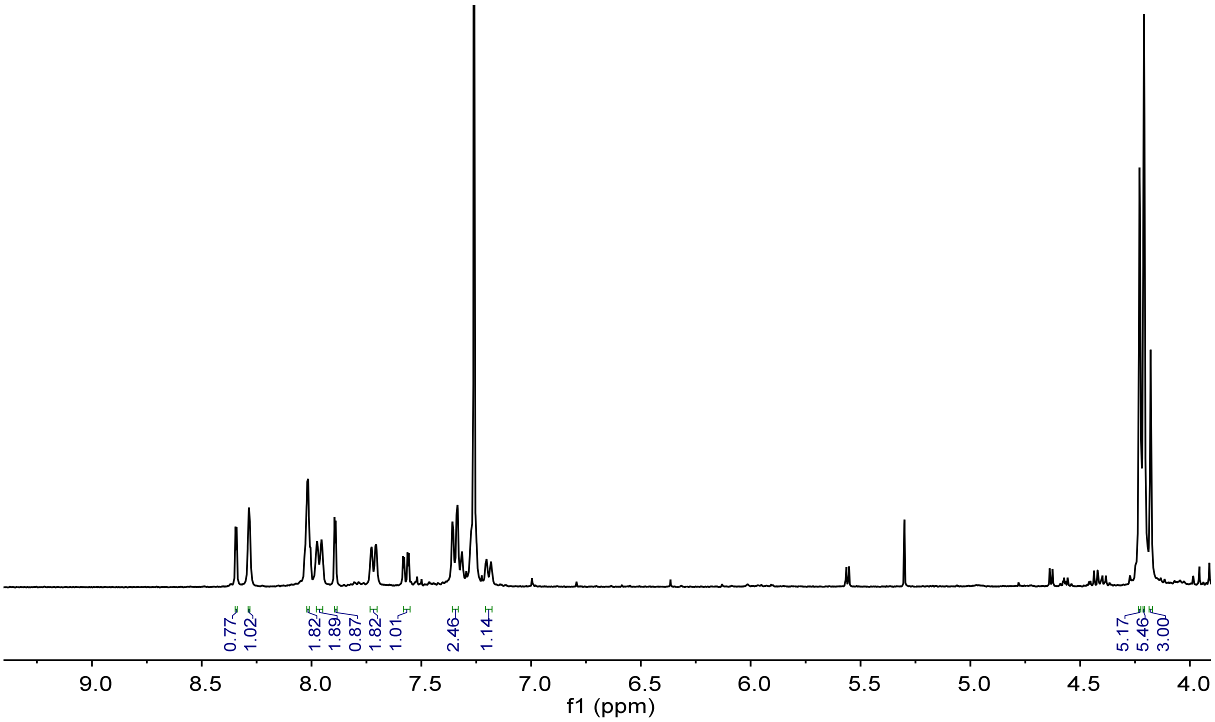

Figure S7. ^{1}H NMR spectrum of Re[Br_{11}T*p*CH_3PC](O) in $CDCl_3$ at 298 K.

b) ESI mass Spectra

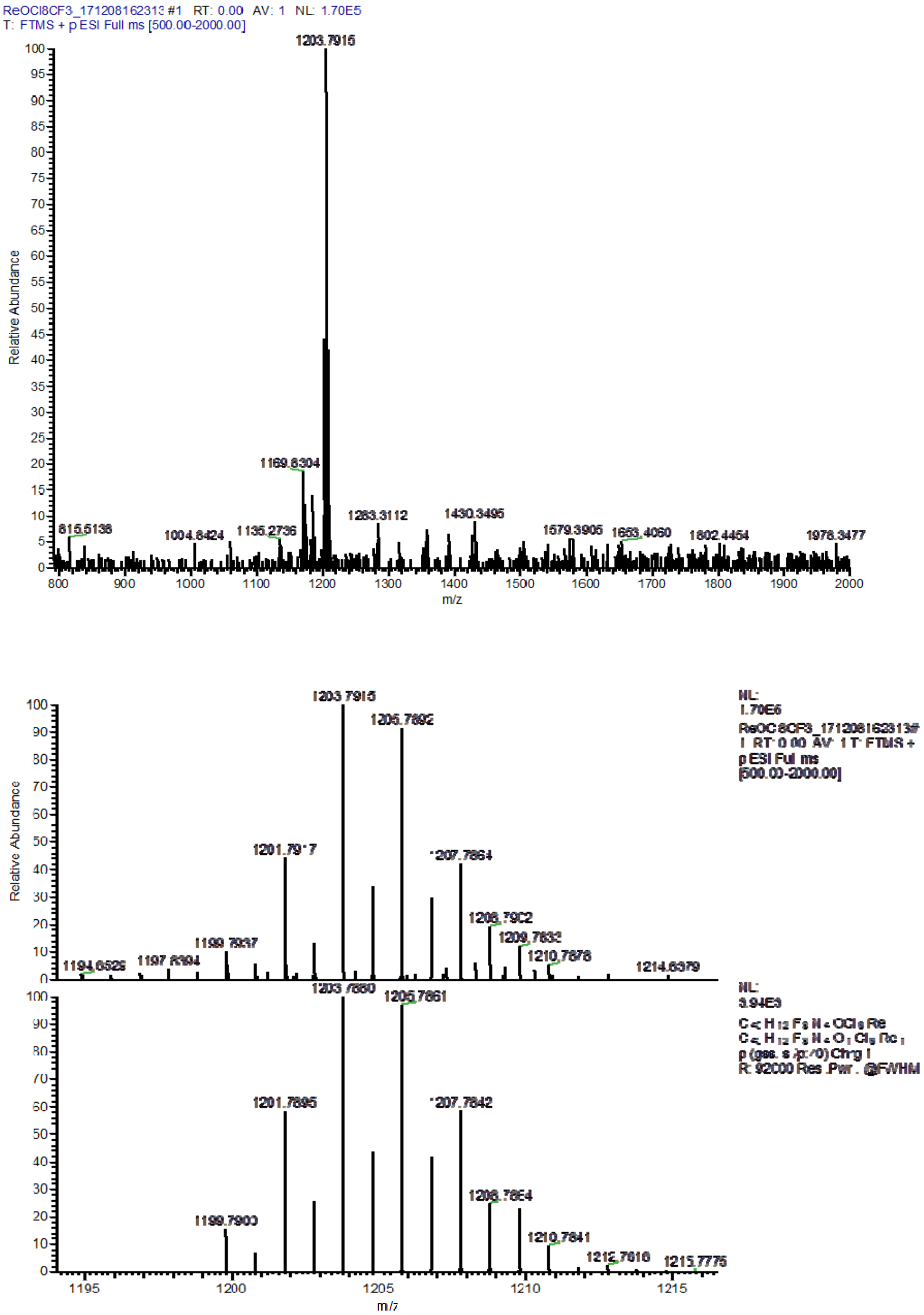

Figure S8. ESI-MS of Re[Cl8T*p*CF3PC](O): experimental (top) and simulation (bottom).

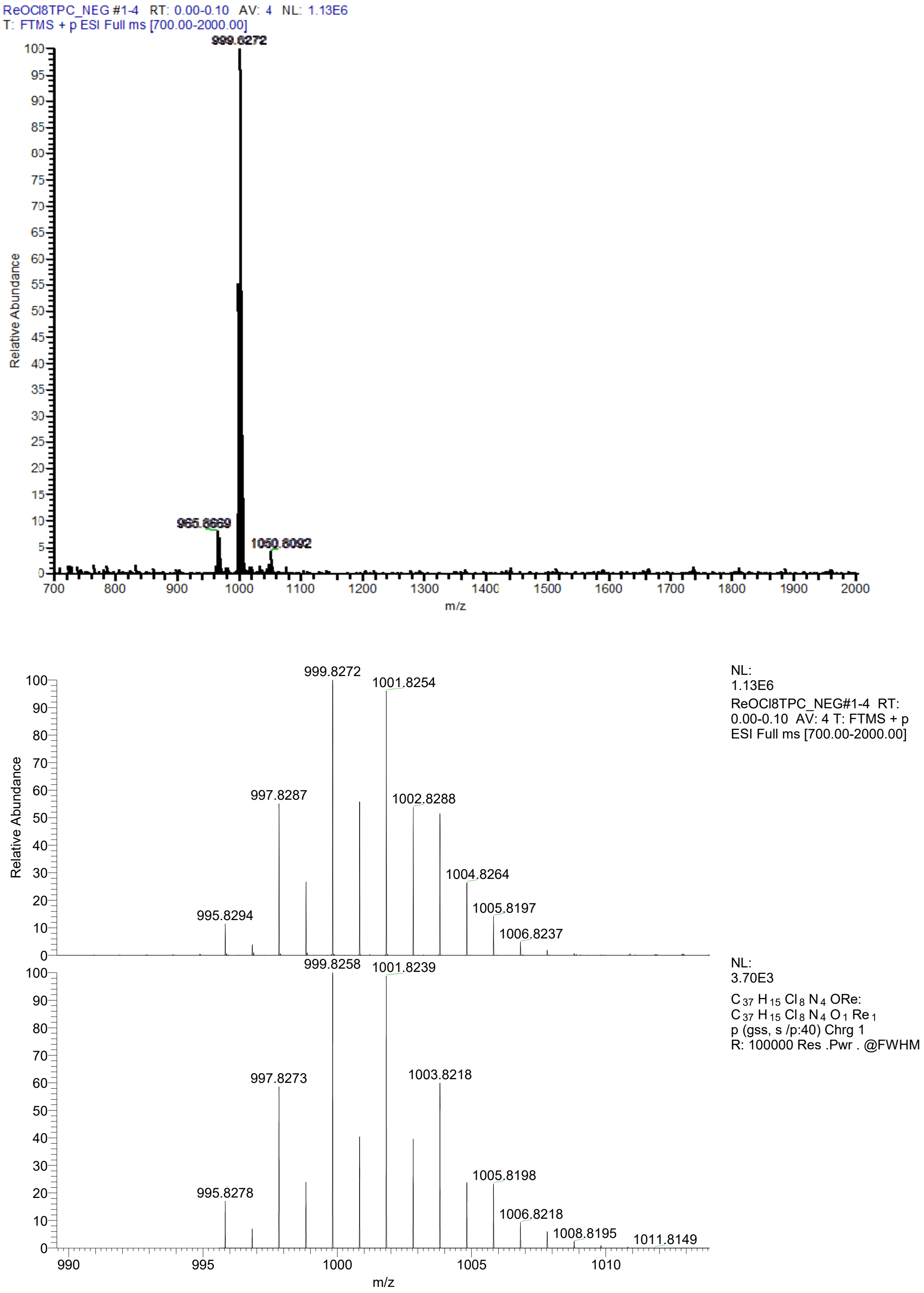

Figure S9. ESI-MS of Re[Cl_8TPC](O): experimental (top) and simulation (bottom).

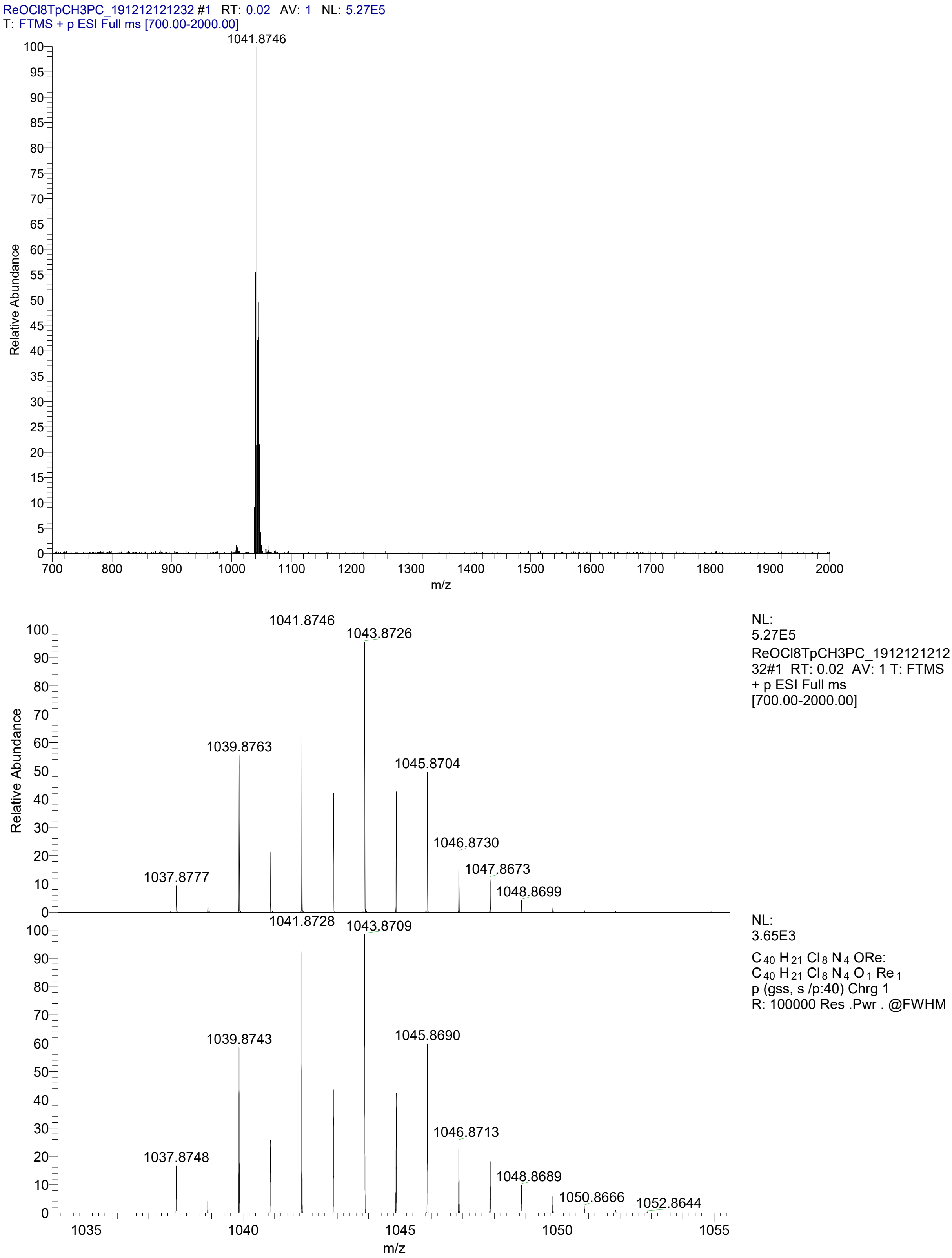

Figure S10. ESI-MS of Re[Cl$_8$T*p*CH$_3$PC](O): experimental (top) and simulation (bottom).

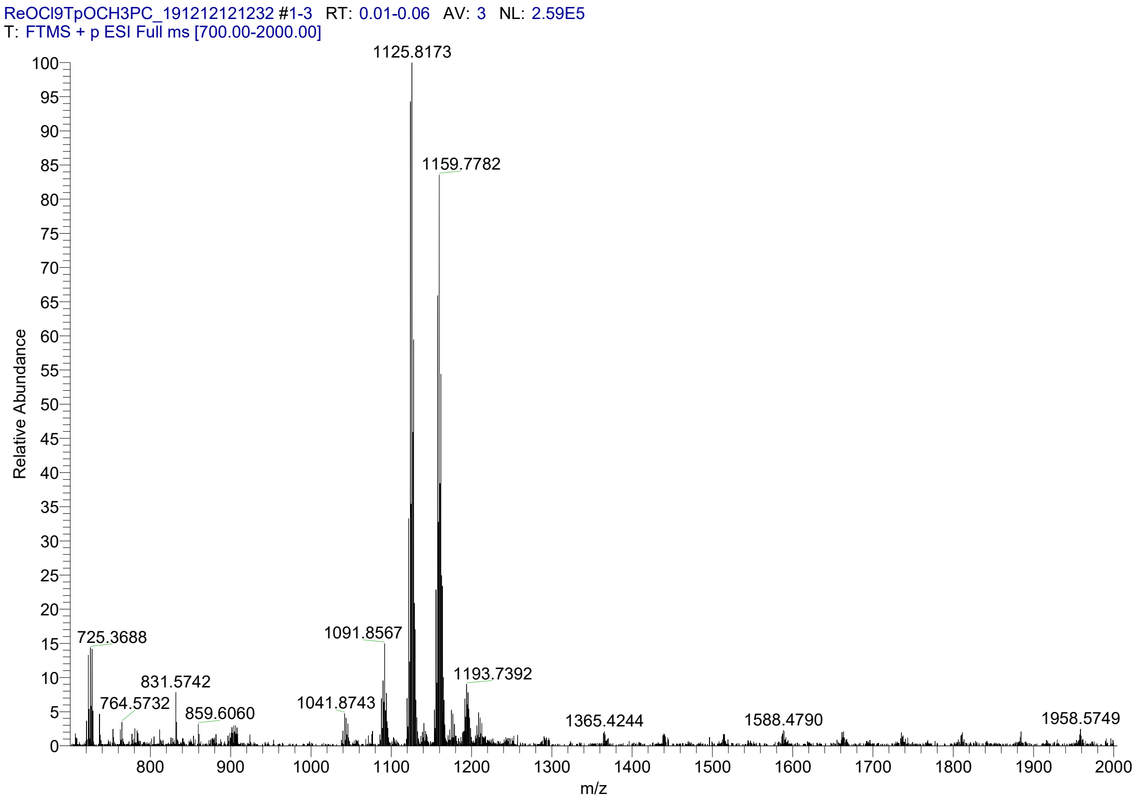

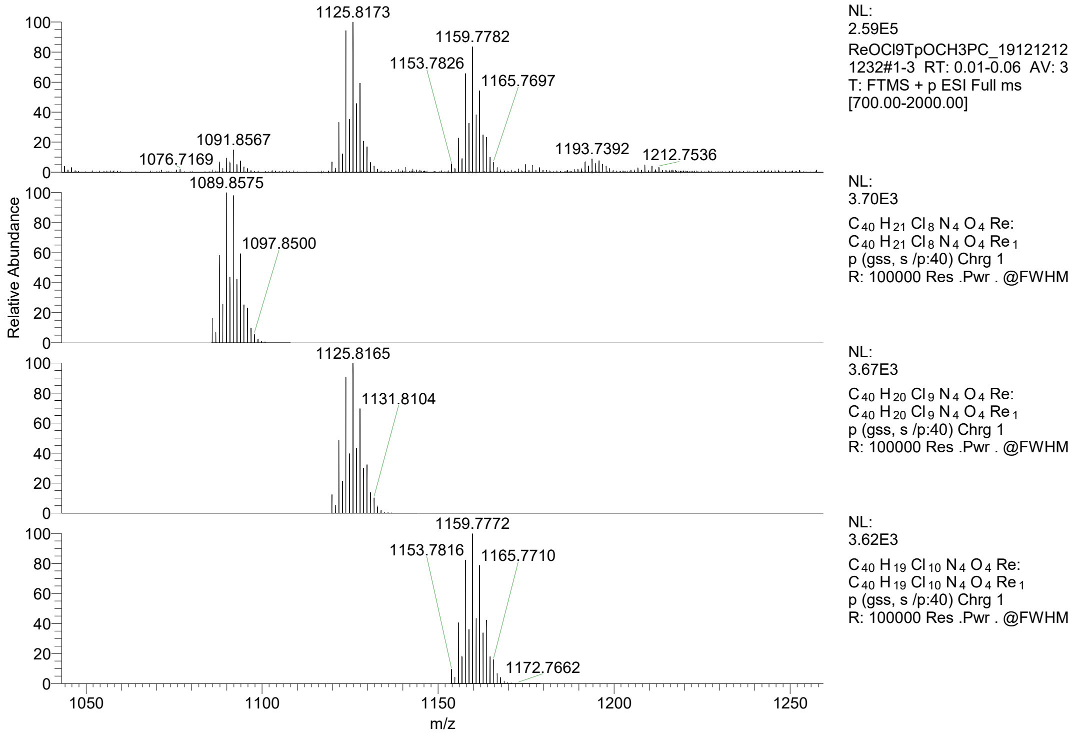

Figure S11. Contd.

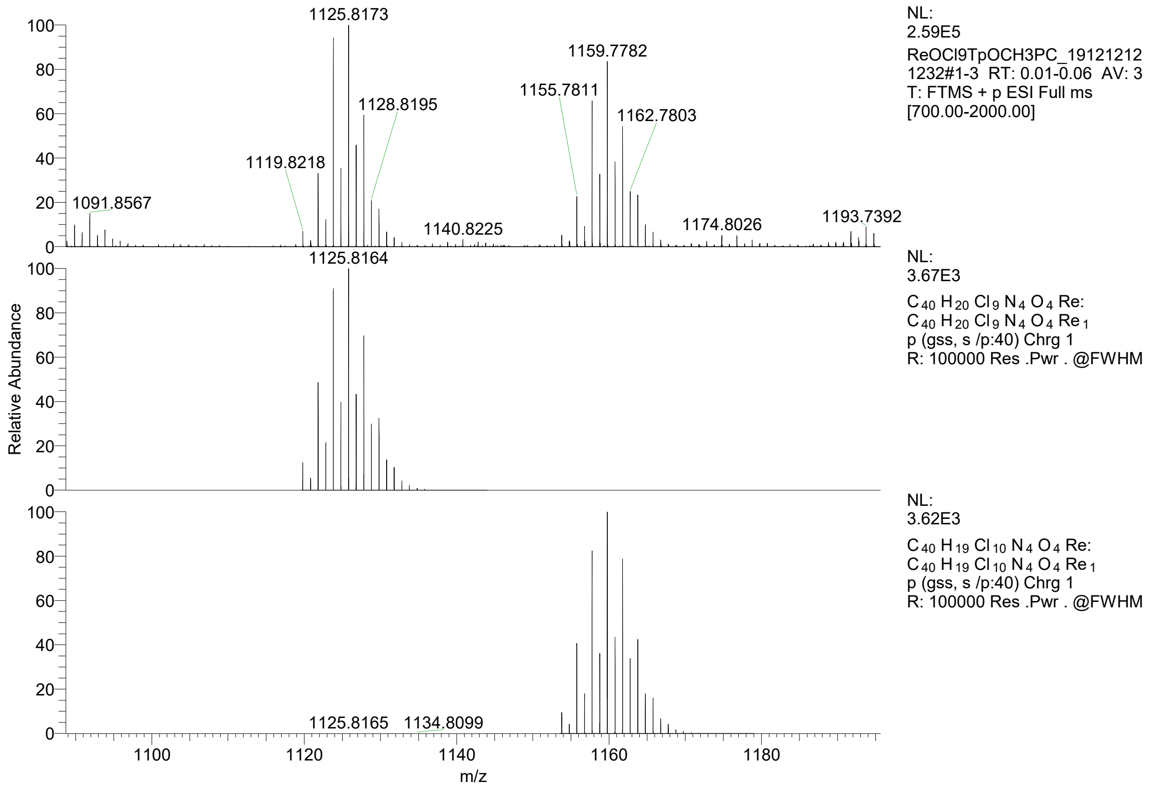

Figure S11. ESI-MS spectrum of Re[Cl_9T*p*OCH_3PC](O) and Re{Cl_{10}T*p*OCH_3PC}(O): experimental (top) and two simulations (bottom).

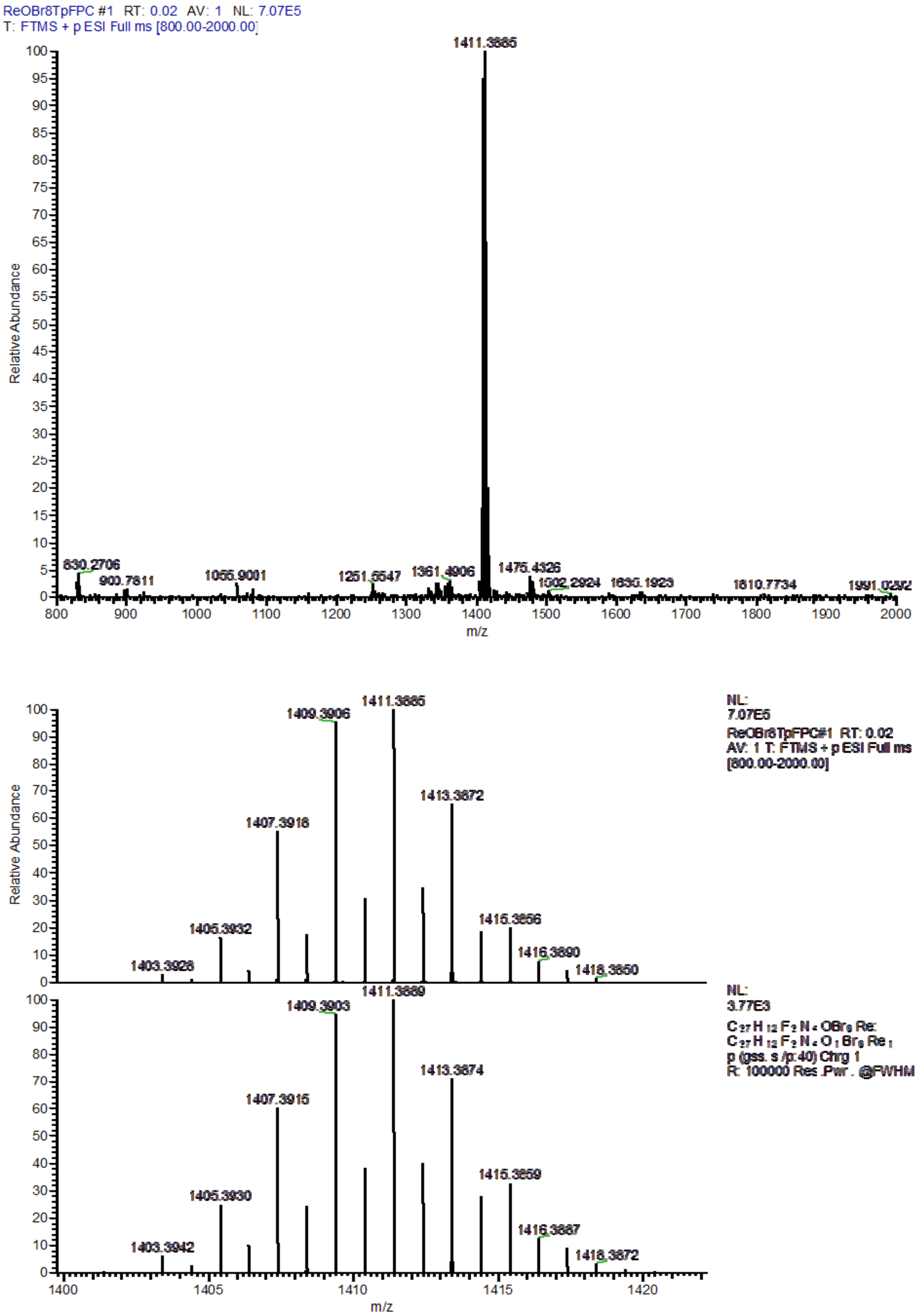

Figure S12. ESI-MS of Re[Br_8T*p*FPC](O): experimental (top) and simulation (bottom).

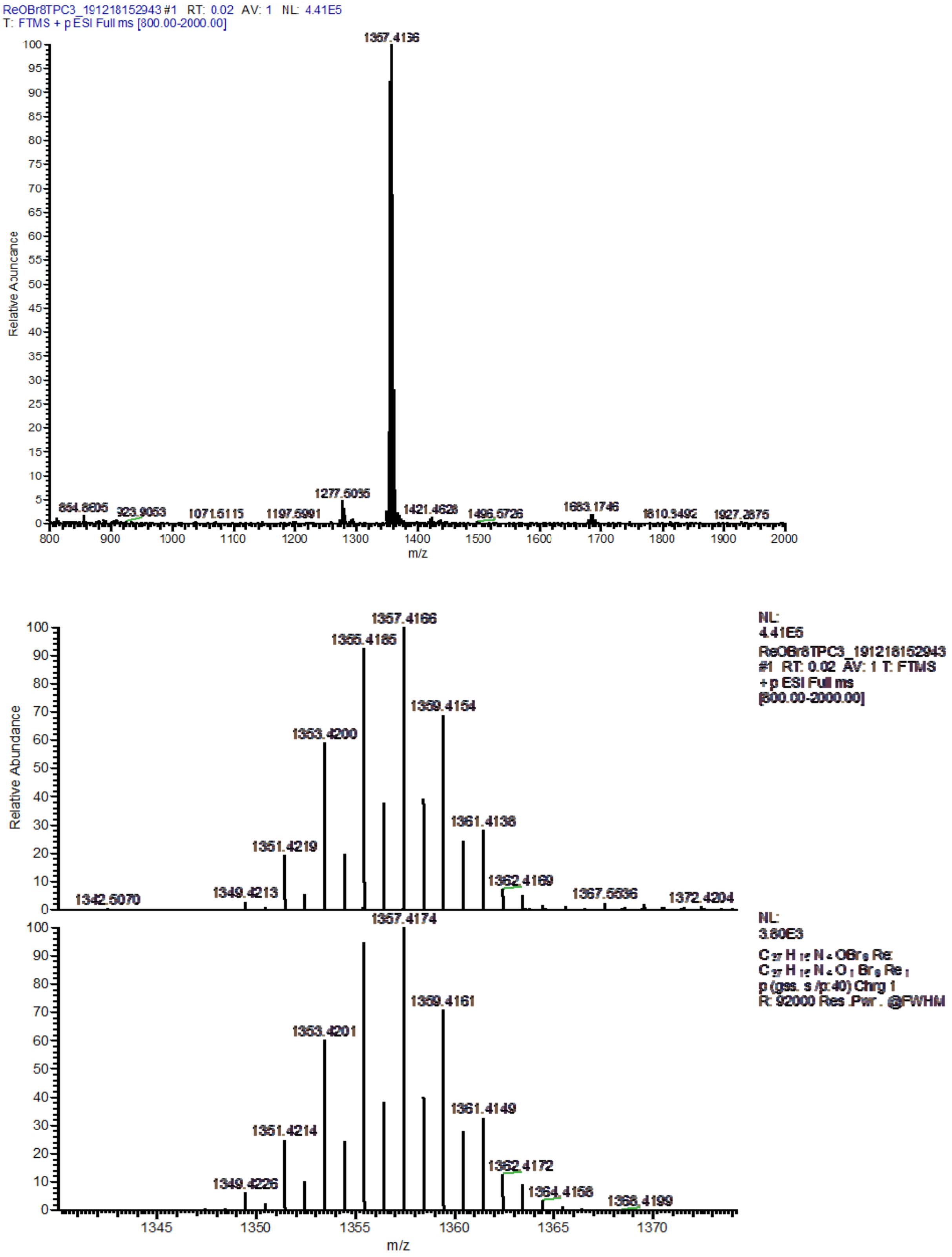

Figure S13. ESI-MS of Re[Br_8TPC](O): experimental (top) and simulation (bottom).

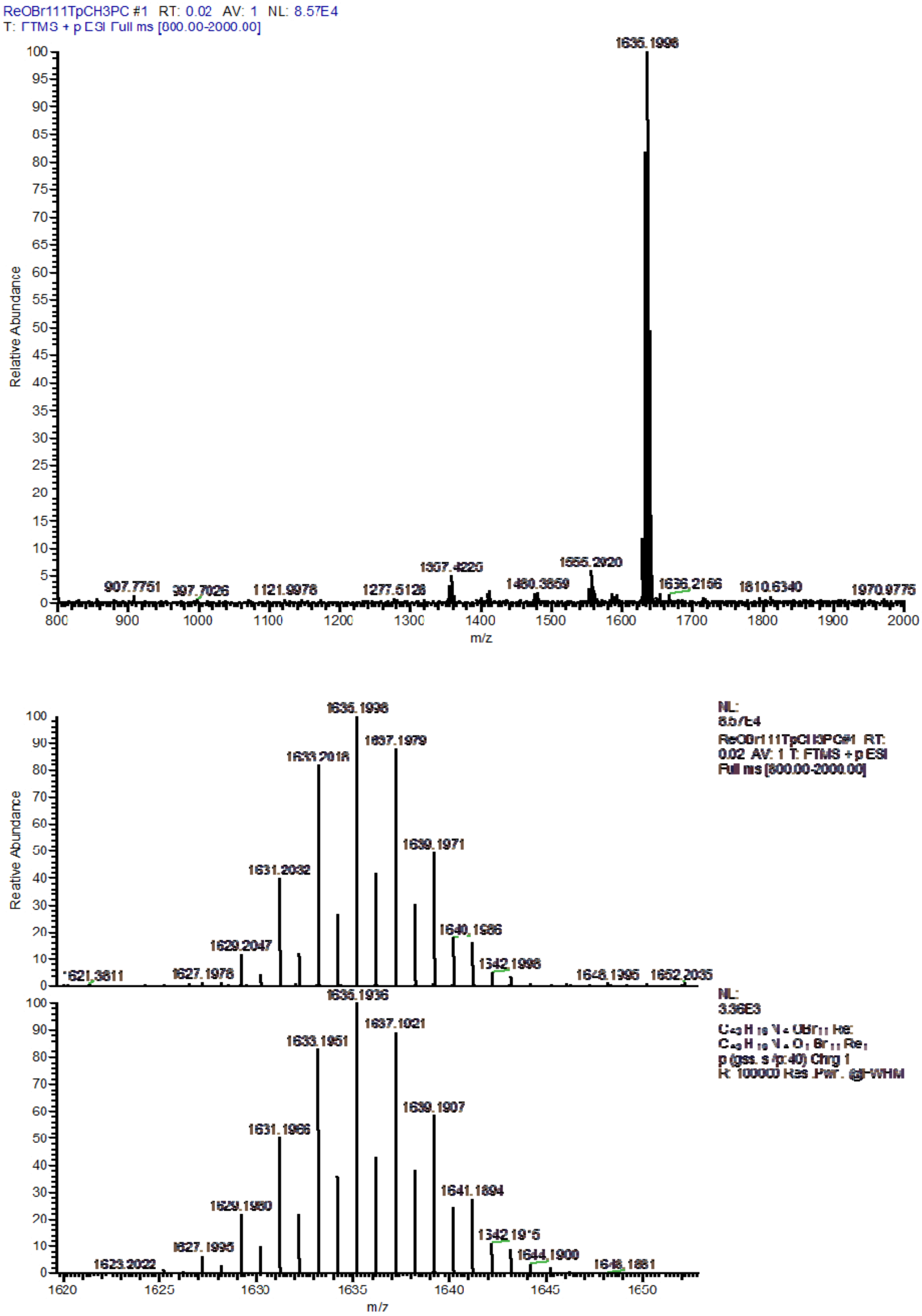

Figure S14. ESI-MS of Re[Br_{11}T*p*CH_3PC](O): experimental (top) and simulation (bottom).

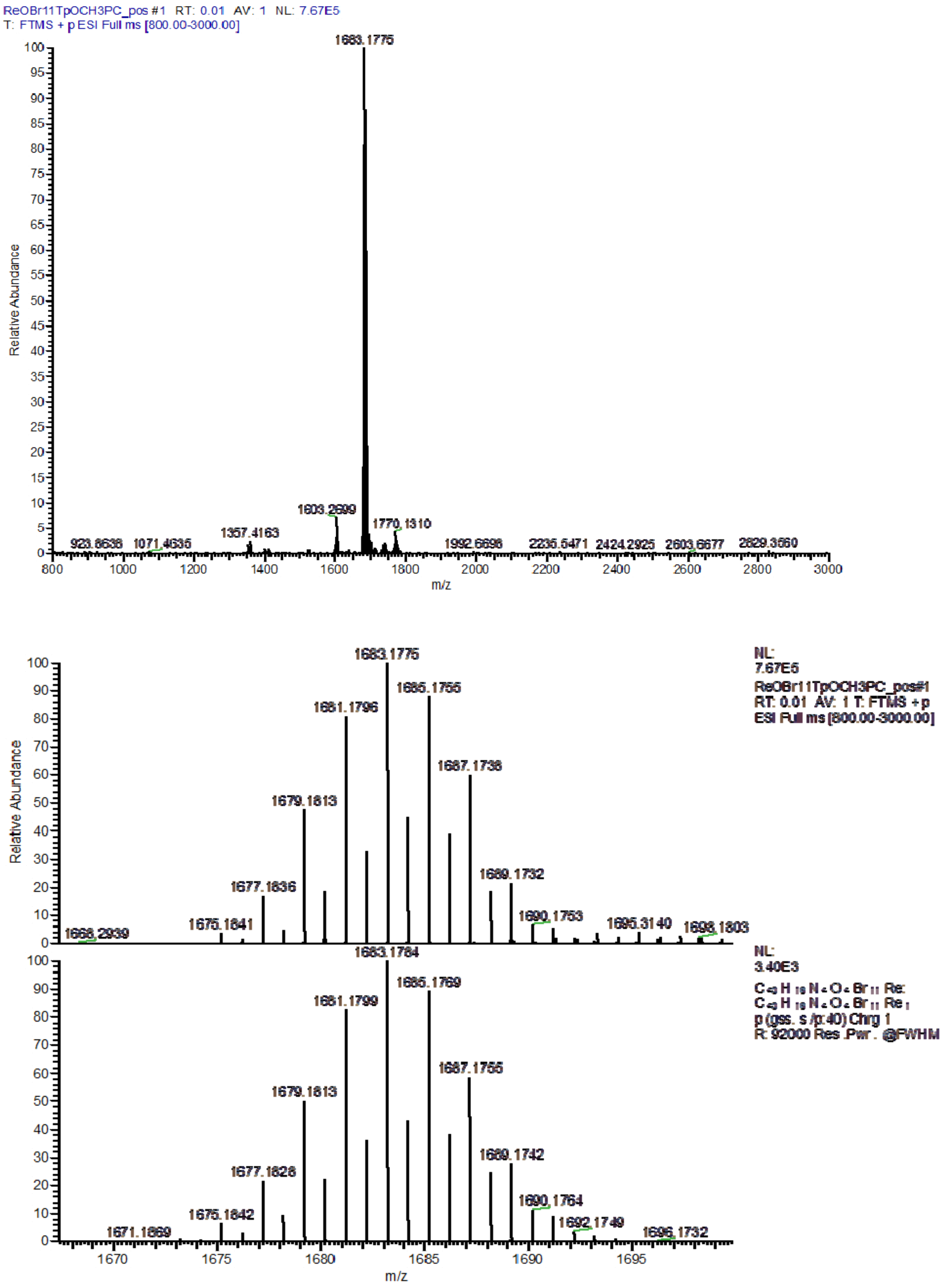

Figure S15. ESI-MS of Re[Br$_{11}$T*p*OCH$_3$PC](O); experimental (top) and simulation (bottom).
